# Tailoring nutrition therapy to illness and recovery

**DOI:** 10.1186/s13054-017-1906-8

**Published:** 2017-12-28

**Authors:** Paul E. Wischmeyer

**Affiliations:** Department of Anesthesiology and Surgery, Duke Clinical Research Institute, Duke University School of Medicine, Durham, NC USA

**Keywords:** Protein, Lean body mass, Muscle, Calories, Critical care, ICU, Quality of life, Recovery, Malnutrition

## Abstract

Without doubt, in medicine as in life, one size does not fit all. We do not administer the same drug or dose to every patient at all times, so why then would we live under the illusion that we should give the same nutrition at all times in the continuum of critical illness? We have long lived under the assumption that critical illness and trauma lead to a consistent early increase in metabolic/caloric need, the so-called “hypermetabolism” of critical illness. What if this is incorrect? Recent data indicate that early underfeeding of calories (trophic feeding) may have benefits and may require consideration in well-nourished patients. However, we must confront the reality that currently ICU nutrition delivery worldwide is actually leading to “starvation” of our patients and is likely a major contributor to poor long-term quality of life outcomes. To begin to ascertain the actual calorie and protein delivery required for optimal ICU recovery, an understanding of “starvation” and recovery from starvation and lean body mass (LBM) loss is needed. To begin to answer this question, we must look to the landmark Minnesota Starvation Study from 1945. This trial defines much of the world’s knowledge about starvation, and most importantly what is required for recovery from starvation and massive LBM loss as occurs in the ICU. Recent and historic data indicate that critical illness is characterized by early massive catabolism, LBM loss, and escalating hypermetabolism that can persist for months or years. Early enteral nutrition during the acute phase should attempt to correct micronutrient/vitamin deficiencies, deliver adequate protein, and moderate nonprotein calories in well-nourished patients, as in the acute phase they are capable of generating significant endogenous energy. Post resuscitation, increasing protein (1.5–2.0 g/kg/day) and calories are needed to attenuate LBM loss and promote recovery. Malnutrition screening is essential and parenteral nutrition can be safely added following resuscitation when enteral nutrition is failing based on pre-illness malnutrition and LBM status. Following the ICU stay, significant protein/calorie delivery for months or years is required to facilitate functional and LBM recovery, with high-protein oral supplements being essential to achieve adequate nutrition.

## Background

### “One size does not fit all”

Without doubt, in medicine as in life, one size does not fit all. We do not administer the same drug or dose of drug to every patient at all times, so why would we live under the illusion that we should give the same nutrition or amount of nutrition at all times? We have long lived under the assumption that critical illness and trauma lead to a consistent early increase in metabolic/caloric need, the so-called early “hypermetabolism” of critical illness and injury. What if this is, and has always been, incorrect? Further, recent data have indicated that early hypocaloric feeding (so-called trophic feeding) may be superior [1, 2]. Could there be some truth to this? Or is the reality that our current ICU feeding practice around the world is actually leading to “starvation” of our patients and is a major contributor to poor long-term quality of life (QoL) outcomes [3]?

Before we can discuss the actual calorie and protein needs of ill and injured patients, what constitutes “starvation-level” nutrition delivery? The reality is, very limited data exist on what constitutes starvation and calorie/protein deprivation, even in healthy individuals. However, one landmark study that very few of us in medicine are ever taught (or even told about) defines much of the world’s knowledge about starvation, and most importantly what is required for recovery from starvation and massive lean body mass (LBM) loss, as commonly occurs in the ICU. This is not a new study, the reality is it was completed > 70 years ago and will almost assuredly never be repeated.

### “The Minnesota Starvation Study—The Most Important and Daring Nutrition Trial Ever Conducted?”

In 1944, as World War II began to draw to a close, many in the USA and around the world began to recognize that the greatest threat to the survival of the world’s population, both for the remainder of the war and after, was not bombs and bullets, but hunger! The war had left hundreds of thousands starving in Europe and Asia, and rebuilding these nations would not be possible with much of the world suffering from a lack of basic nutrition. US soldiers entering liberated European cities found emaciated, cachectic, and starved civilians surviving on meager portions of potatoes, bread, and little more. At that time, very little knowledge existed about the fundamental nutritional needs in humans. Thus, the USA and other nations wishing to support relief efforts worldwide realized a greater understanding of how to deal with refeeding and the nutrition delivery required to recover from severe starvation was desperately needed. How else would nations supplying the life-saving food relief know how much was needed to ensure recovery?

As a result, Dr Ansel Keys, a young physiology professor at the University of Minnesota and a consultant to the War Department, set out to assess how civilians would be affected physiologically and psychologically by such a limited diet and what would be the most effective way to provide postwar “nutritional rehabilitation” [4]. As a result, he and a small group of scientists conceived one of the most ambitious and important human clinical trials in history— the “Minnesota Starvation Study” [5]. (For further details, see the excellent summary by Kalm and Semba [6]).

**Fig. 1:**
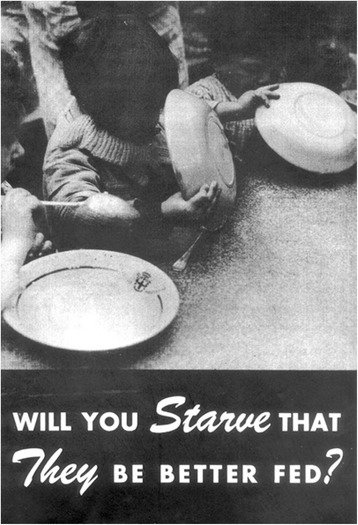
Minnesota Starvation Study recruitment brochure from May 27, 1944. Adapted from [6]

As the US involvement in World War II grew, many young men (and women) enlisted in the military. However, due to religious beliefs, morals, or conscience some chose not to fight. These individuals became known as conscientious objectors (COs)—COs were commonly sent to do menial jobs like building roads, forestry work, and other peaceful homeland contributions. However, in 1944 Keys gave a few heroic COs a chance to contribute in a legendary way. Keys obtained approval from the War Department to find healthy men from the 12,000 COs registered across the country. The men had responded to a recruitment brochure that asked: “Will You Starve That They Be Better Fed?” (Fig. 1). Within months Keys received > 400 positive responses and 100 men were brought in for interviews and screening physical examinations. After extensive screening and explanation of the trial, 36 subjects were selected for the study. As with most great scientific and medical endeavors, this experiment was jointly funded by the government (Office of the Surgeon General), foundational support (from religious groups including Mennonites, Brethren, Quakers, and Unitarians), and private industry funding. Thus, on November 19, 1944, 36 healthy young men entered the brick confines of the Laboratory of Physiological Hygiene, located in the South Tower of the football stadium at the University of Minnesota. The laboratory also served as their dormitory, and the windowless rooms of the laboratory were often referred to by Keys as “our cage” [5].

The “experiment” consisted of a 3-month baseline period in which subjects received 3200 kcal/day and participated in regular physical activity. Extensive physiologic, cognitive, intelligence, and laboratory testing was conducted throughout the experiment. A 6-month “semi-starvation” period, beginning on February 12, 1945, delivered a “starvation diet” of on average 1800 kcal of food/day with 0.7–0.9 g/kg/day of protein—considered a “low protein diet”. During the semi-starvation period, subjects initially consumed an average of 23 kcal/kg/day with a protein intake of 0.7 g/kg/day, with a plan for the subjects to lose ~ 25% of their body weight (~1.0 kg/week) by the end of the study period. Although the absolute amount of energy and protein consumption was fairly constant during the semi-starvation period, weight loss was occurring too rapidly in many subjects and by the end of the study the average intake per kilogram had increased to 30 kcal/kg/day and 0.9 g protein/kg/day, with significant starvation persisting at these energy delivery levels. The starvation diet was created to consist of foods reflecting the diet experienced in the war-torn areas of Europe (i.e., potatoes, turnips, rutabagas, bread, etc.).

**Fig. 2:**
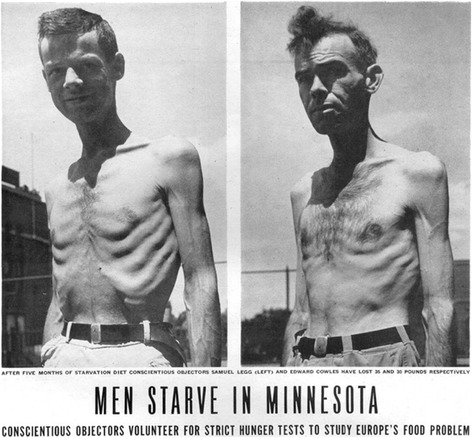
Photograph from *Life* magazine on July 30, 1945 (volume 19, number 5, p. 43) showing men enrolled in the Minnesota Starvation Study during the semi-starvation diet. Adapted from [6]

The effects of the semi-starvation diet were quick and striking. Men in the study lost weight rapidly and all men developed significant edema from protein malnutrition. Subjects rapidly demonstrated a remarkable decline in strength and energy. Keys recorded a 21% reduction in their strength, as measured by performance on a back-lift dynamometer. All subjects complained that they felt old and constantly fatigued. Significant depression, anxiety, neurologic deficits, and loss of interest in sex occurred. Men become obsessed with food and cheating on the diet became an issue. Thus Keys began a buddy system to improve compliance in which no one was allowed out alone (“buddy system”). The stress proved too much for one of the men, 24-year-old subject Franklin Watkins (as described online: http://www.madsciencemuseum.com/msm/pl/great_starvation_experiment). He began having vivid, disturbing dreams of cannibalism in which he would consume the flesh of an old man. On trips into town, before the buddy system had been implemented, he was known to cheat extravagantly on the starvation diet, downing milkshakes and ice cream. Finally, Keys confronted him, and Watkins broke down crying. Watkins then became agitated and threatened to kill Keys and take his own life. Keys immediately dismissed Watkins from the study and had him admitted to the psychiatric ward of the university hospital. There, after a just a few days on a normal diet, Watkins’ cognition and mood fully normalized, and he was released from the hospital. Strikingly, Watkins’ breakdown occurred just a few weeks into the starvation phase of the experiment. This study received a great deal of national attention, including a prominent depiction in *Life* magazine in July 1945 (Fig. 2).

By the end of the 6-month starvation period, the men had lost almost a quarter of their weight, dropping from an average of 152.7 lb (70 kg) down to 115.6 lb (52 kg). The average heart rates of the subjects slowed dramatically, from an average of 55 to 35 beats per minute. Their blood volume dropped 10%, and their hearts shrank in size. The last day of the starvation period (July 28, 1945) was met with great enthusiasm and anticipation by the men.

However, July 29, 1945, did not prove to be the reprieve they had anticipated. The final 3 months of the study consisted of a structured “nutritional rehabilitation” period. Keys divided the men into four subgroups, with each receiving an additional 400, 800, 1200, or 1600 kcal/day respectively above the amount of food delivered in the starvation phase, leading to a total of 2200–3400 kcal/day. Unfortunately, this increase in caloric delivery did not improve the men’s starvation state! Very little appreciable weight gain occurred in any of the groups and some men continued to lose weight on the increased calorie diets. This led Keys to further increase the men’s caloric delivery by 800 kcal/day in each group. This led to a 1200–2400 kcal/day increase per group for a total of 3000–4200 kcal/day. This finally led to successful weight gain in the starving men. To attempt to assist post-war relief efforts, Keys released early results related to the most effective of the various rehabilitation diets before the experiment even ended [7, 8]. At a 1945 scientific meeting in Chicago, Keys noted:

Enough food must be supplied to allow tissues destroyed during starvation to be rebuilt … our experiments have shown that in an adult man no appreciable rehabilitation can take place on a diet of 2000 calories [actually 2000 kcal] a day. The proper level is more like 4000 [4000 kcal] daily for some months.

The study officially ended on November 20, 1945. Keys convinced 12 of the men to stay on in the study for another 8 weeks so that he could monitor them during an “unrestricted nutritional rehabilitation” phase. Able to consume food at will, Keys observed that the men consumed an average of over 5000 calories/day. Some of the men were noted to take in as much as 11,500 calories in a single day! For many months, the men reported having a sensation of hunger they could not satisfy, no matter how much they ate. In these fully healthy, young men, recovery to a normal weight took an average of between 6 months and 2 years. No appreciable long-term or permanent adverse effects were noted in the subjects. This work led to the landmark two-volume, 1385-page publication *The Biology of Human Starvation* in 1950 [5].

### Can we learn from the Minnesota Starvation Study how to provide “goal-directed” and targeted feeding in illness and recovery?

One of the first and most striking lessons from this study and others since is the amount of calories and protein a normal, healthy individual requires to maintain body weight and physical/mental function. Remember the initial caloric delivery in the control period of the Minnesota Study was 3200 kcal/day. This seems excessive as we think of the obesity epidemic and excess of caloric intake often present in the First World (clearly not true in many developing countries); however, based on the World Health Organization (WHO) and the Food and Agriculture Organization of the United Nations, this is not far from current WHO recommendations. Current data presented in Table 1 indicate that for a moderately active 70-kg individual (1.75 × BMR) between the ages of 30 and 60 the daily energy requirement (or approximate total energy expenditure (TEE)) is 3000 kcal/day (44 kcal/kg/day) for men and 2500 kcal/day (36 kcal/kg/day) for women (http://www.fao.org/docrep/007/y5686e/y5686e00.htm#Contents). The recommended WHO baseline protein delivery to avoid starvation in humans is ~ 0.75 g/kg/day. Interestingly, this calorie delivery is virtually identical to the control period of the Minnesota Study.

**Table 1:** Summary of caloric needs of critically ill and healthy individuals in the context of the Minnesota Starvation Study and actual current ICU calorie delivery

	Mean REE (kcal/day)	TEE (kcal/day)	TEE/weight (kcal/kg/day)
Uehara et al., ICU study [12]			
Sepsis patients (mean age 67)			
Week 1	~ 1854	1927 ± 370	25 ± 5
Week 2		3257 ± 370	47 ± 6
Trauma patients (mean age 34)			
Week 1	~ 2122	2380 ± 422	31 ± 6
Week 2		4123 ± 518	59 ± 7
WHO calorie requirements, healthy subjects^a^			
Men		~ 3000	44 (range 35–53)
Women		~ 2500	36 (range 29–44)
Minnesota Starvation Study calorie delivery		Delivered energy (kcal/day)	Delivered energy/weight (kcal/kg/day)
Baseline period		3200	~ 50
Starvation period		~ 1800	23–30
Recovery period delivery (for recovery to occur)		~ 4000	~ 60

Actual average 1034 kcal/day delivered in critically ill patients over first 12 days of ICU stay [15]

*REE* resting energy expenditure, *TEE* total energy expenditure, *WHO* World Health Organization

^a^Data for a healthy 70-kg person with intermediate physical activity (1.75 physical activity level factor). Reference: http://www.fao.org/docrep/007/y5686e/y5686e00.htm#Contents

**Fig. 3:**
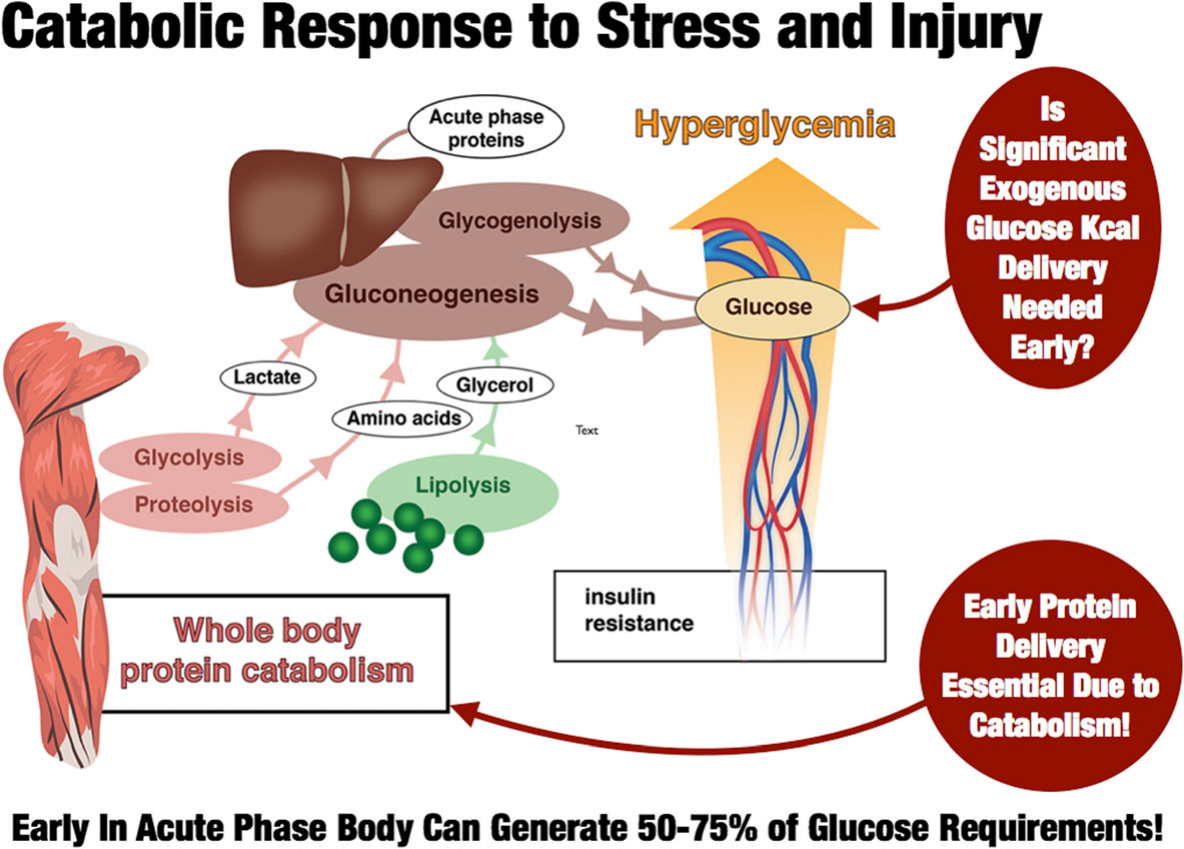
Substrate mobilization in catabolic response to stress and injury during acute phase. In well-nourished patients, the body is capable of generating 50–75% of glucose needs in the first few days of ICU stay. Patients still require adequate protein delivery (> 1.0 g/kg/day) due to muscle catabolism, but may benefit from reduced nonprotein kilocalorie delivery (~ 15 kcal/kg/day). Adapted from [9]

**Fig. 4:**
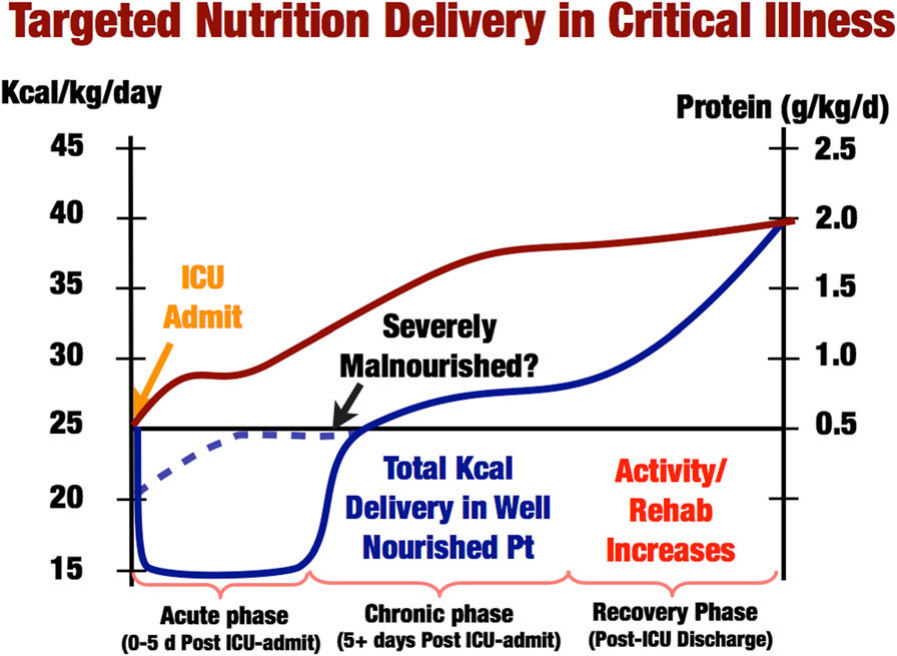
Proposal for targeted nutrition delivery across phases of critical illness. Adapted from [18]

As we begin to examine how to deliver targeted calorie and protein delivery based on actual physiologically measured targets in critical illness, we must examine the existing data for caloric need in the different phases of critical illness. “Targeted” nutrition delivery emphasizes that we should take into account that long-standing basic metabolism data showing nutritional needs can change significantly over the course of critical illness. It is well described that the early or “acute phase” of critical illness is characterized by massive mobilization of the body’s calorie reserves as muscle, glycogen, and lipid stores are broken down to drive glucose production [9, 10] (see Fig. 3). This evolutionarily conserved response allows the stressed or injured human to generate energy to escape its attacker and recover from initial injuries. This metabolic response to stress can generate 50–75% of glucose needs during illness [10], and this glucose generation is not suppressed by feeding or intravenous glucose infusion [11]. This is described in much greater detail by Oshima et al. [11] with recent data from our group. Further, we know that the early acute phase of sepsis and trauma are not hypermetabolic states, but rather the patients have a TEE to resting energy expenditure (REE) ratio of 1.0 and 1.1 for sepsis and trauma respectively [12]. Thus, caloric need does not increase in the early phases of injury (first few days post injury). In fact the more severe the septic shock, the lower the resting energy, as the body “hibernates” and shuts down metabolism in response to severe stress [13]. As presented in Table 1, data from Uehara et al. [12] show us that the REE in the first 2–5 days (acute phase) in elderly sepsis patients (mean age 67) is ~ 1850 kcal/day with a TEE of ~ 1920 kcal/day for a TEE of 25 kcal/kg. In the 2nd week following sepsis this increases to a TEE of ~ 3250 kcal/day or 47 kcal/kg/day—virtually identical to WHO requirements for normal, healthy humans. In younger trauma patients (mean age 34), Uehara et al. described an even greater increase in caloric need in the 2nd week post injury to an average of ~ 4120 kcal/day or 59 kcal/kg/day, nearly identical to the 4000 kcal/day that Keys demonstrated was required to recover from starvation in the young subjects in Minnesota. This demonstrates that in the later recovery phase of critical illness, the body experiences a massive increase in metabolic needs, with TEE increasing as much as ~ 1.7-fold above REE [12]. With the onset of early ICU mobility programs, this may increase further as activity increases. Thus, as presented in Table 2, sources of energy supply transition in critical illness from largely endogenous supplies and release of energy early in illness to the need for primarily exogenous energy delivery in the late or recovery phase [11]. These data suggest we should consider feeding less nonprotein calories early in the acute phase (first 24–96 hours) of critical illness and markedly increase calorie delivery during recovery as illustrated in Fig. 4. Further, new data indicate that thiamine deficiency occurs in up to 35% of septic shock patients [14]. A recent randomized, double-blind, controlled trial administered 200 mg thiamine to patients with septic shock and elevated lactate [14]. Administration of thiamine did not improve lactate levels or other outcomes in the overall group of patients with septic shock and elevated lactate. However, in thiamine-deficient patients, a statistically significant decrease in mortality over time in those receiving thiamine was observed (*p* = 0.047), as well as reduced lactate at 24 hours [14].

**Table 2:** Conceptual transitions of utilization of energy supply in acute illness

Utilization of energy source	Phase of critical illness		
	Acute	Chronic	Post-acute
Endogenous	Maximal	Reduced	Marginal
Exogenous	Minimal	Increasing	Maximal

Adapted from [11]

At the same time, it is also well known that protein losses increase 4-fold in the first 24 hours of critical illness [15] and we are exceedingly poor at meeting these needs [15]. Unfortunately, large, international surveys indicate that we as ICU practitioners deliver an average of 0.6 g/kg/day of protein for the first 2 weeks following ICU admission [16]. This is one-third to one-half of the latest ICU guideline-recommended protein delivery of 1.2–2.0 g/kg/day [17]. In contrast to what is often taught, the delivery of additional nonprotein calories does not significantly improve the nitrogen balance in illness beyond delivery of 50% of predicted REE. Thus, an ideal “targeted” feeding strategy is perhaps ~ 15–20 kcal/kg/day of total energy during the early ICU stay (acute phase), while ensuring patients receive adequate protein delivery (1.0–1.2 g/kg/day) as early as possible post ICU admission [18] (Fig. 4). Reduced calorie delivery during the acute phase is likely not applicable in malnourished patents (i.e., patients with significant pre-ICU weight loss or NUTRIC Score (w/o IL-6) > 5) who are unlikely to have the metabolic reserve to generate needed endogenous energy [17, 19]. Ironically, our most recent SCCM/ASPEN Guidelines emphasize these points in updates suggesting hypocaloric PN (≤ 20 kcal/kg/day or 80% of estimated energy needs) with adequate protein (≥ 1.2 g protein/kg/day) should be considered in patients requiring PN over the first week in the ICU [17]. Further, in early sepsis (or the acute phase of critical illness) the new SCCM/ASPEN Guidelines suggest provision of trophic feeds (defined as 10–20 kcal/hour up to 500 kcal/day) for the initial phase of sepsis, advancing as tolerated after 24–48 hours to > 80% of target energy with early delivery of 1.2–2 g protein/kg/day [17].

### Is it possible we already “hypocalorically” feed our ICU patients far beyond the acute phase?

Extensive data for international ICU nutrition delivery currently exist from the International Nutrition Survey, which is conducted regularly by the Canadian Critical Care Nutrition Group (www.criticalcarenutrition.com). These data reveal that the average for calories delivered in the ICU over the first 12 days is 1034 kcal and 47 g of protein (Table 1) [16]. This period is far longer than the first 1–5 days of the acute phase where hypocaloric feeding (with adequate protein) may make physiologic sense. In fact, more troubling, this total is far lower than the 1800 kcal/day and ~ 0.8 g/kg/day which led to severe starvation in the Minnesota Starvation Study! Thus, in comparison, nutrition delivery in the ICU versus Key’s Starvation Study is as follows: Minnesota Starvation Study (starvation period), 1800 kcal/day and 0.75–0.8 g/kg/protein; and ICU patients worldwide for the first 12 days in the ICU, 1034 kcal/day and 0.6 g/kg/protein.

These data confirm that ICU patients worldwide average far less energy and protein than in the legendary Minnesota Starvation Study, a study that would likely never be repeated today due to questions around the ethics of inducing potentially life-threatening starvation in a healthy volunteer. Yet it appears to be quite acceptable to actively starve ICU patients worldwide, and to a much more severe degree then the men in Minnesota suffered (which drove many of the men nearly to the point of insanity)*.* Further, we know that starvation in humans leads to active slowing of metabolism and reduced catabolism of protein over time. Unfortunately, after the first week in the ICU we know that critical illness leads to significant hypermetabolism and severe ongoing protein losses. Moreover, we know that 30–50% of patients are malnourished at hospital admission (unlike the well-nourished men in Key’s Starvation Study), greatly increasing the risk of ongoing inhospital starvation in our ICU patients. Thus, how can we justify the magnitude of starvation we inflict upon our patients daily in our ICUs? Is this not some of the explanation for the increasing number of ICU survivors who ultimately become “victims” of post-ICU syndrome (PICS), never to walk again or return to a meaningful QoL post ICU discharge [20, 21]?

Again we must ask, are we creating survivors, or are we creating victims with the starvation we daily allow to occur in our ICUs?

### How can we improve the worldwide epidemic of starvation in ICU patients?

The basic metabolism and physiology of human nutritional needs described indicate that early hypocaloric feeding in the first few days (acute phase) of critical illness would need to be accompanied by adequate protein delivery to help account for marked protein losses early in the ICU stay. Unfortunately, given the limited high-protein, lower-kilocalorie enteral feeding options available commercially, TPN or enteral protein supplements will currently be required to achieve this in most cases. TPN is now a significantly more viable option to achieve this as three recent large trials of both supplemental and full TPN support versus EN in the ICU setting have shown that TPN use in the ICU is no longer associated with increased infection risk [22–24]. This is likely due to improvements in glucose control, central line infection control measures, and potentially as a result of improved (nonpure soy-based) lipid formulations as described in detail in the recent review by Manzanares et al. [25]. In support of early TPN use, the new SCCM/ASPEN Guidelines indicate that for any patient at high nutrition risk (NRS 2002 > 5 or NUTRIC Score (w/o IL-6 score) > 5) or found to be severely malnourished when EN is not feasible, exclusive PN should be initiated as soon as possible following ICU admission [17].

A subsequent question that must continue to be addressed for the future of critical care is whether achieving goal energy delivery (kcal/day) or just achieving goal protein early during the ICU stay is more essential to outcome. Recent data from Nicolo et al. [26] examined this question and found that only achieving > 80% of protein goals by ICU day 4 or ICU day 12 improved 60-day mortality. Achieving energy goals at day 4 and day 12 was not associated with a statistically significant improvement in mortality outcomes. However, many experts are calling for post-ICU QoL, not survival, to be the most important outcome we should focus on in future ICU outcome trials [27]. When examining the effect of nutrition delivery on post-ICU QoL, Wei et al. [28] recently showed in patients requiring mechanical ventilation for > 8 days that for every additional 25% of goal calories/protein delivered over the first 8 days of the ICU stay, QoL was improved in a number of SF-36 physical function scores and this effect was most significant in the medical ICU patients studied. Thus, avoiding the frequent starvation that plagues our ICU patients in the first 1 or 2 weeks may markedly improve their QoL many months later. This is reinforced by data showing that delivery of greater than 1.0–1.2 g/kg/day of protein seems to be a minimum requirement for nutrition to show a benefit on outcome in the ICU setting [11, 29]. Finally, our recently published TOP-UP trial of supplemental parenteral nutrition in high malnutrition risk patients shows a promising trend in QoL measures for supplemental PN toward improved hospital discharge Barthel Functional Index (*p* = 0.08), handgrip strength (*p* = 0.14), and 6-minute walk test (*p* = 0.2) [30]. This requires further study and QoL measures need to be emphasized as future endpoints of ICU nutrition trials.

### Should all patients receive hypocaloric high-protein feeding in the acute phase: role of pre-existing malnutrition?

Reduced calorie delivery during the acute phase is likely not applicable in malnourished patents (i.e., patients with significant pre-ICU weight loss or NUTRIC Score (w/o IL-6) > 5) who are unlikely to have the metabolic reserve to generate the needed endogenous energy [17, 19]. The NUTRIC Score may be the best and most useful marker to discern patients who are candidates for early high-protein, hypocaloric feeding in the acute phase and which patients are at great nutritional risk and should be started on ~ 25 kcal/kg/day shortly after admission. Patients with a NUTRIC Score (w/o IL-6) > 5 have been shown in both the original trial and in a number of validation trials (i.e., [31]) to benefit most from early goal-oriented (> 80% energy goal) feeding. Thus, these data would suggest that these patients should not receive early hypocaloric feeding given their severe nutrition risk. As the new SCCM/ASPEN Guidelines indicate in patients found to be significantly malnourished (i.e., nutrition risk in critically ill patients with NUTRIC Score (w/o IL-6) > 5 or Nutrition Risk Score (NRS) > 5), when EN is not feasible a recommendation is made for initiating exclusive PN as soon as possible following ICU admission.

### Targeted nutrition in the recovery phase? Significantly increased protein and calorie needs

As the patient enters the recovery phase, total protein and calorie delivery needs to increase significantly as suggested in Fig. 4. As data from the landmark Minnesota Starvation Study [5, 6] demonstrate, a healthy 70-kg human, following significant weight loss, requires an average of 4000–5000 kcal/day for between 6 months and 2 years to fully regain lost muscle mass and weight [5]. As many ICU patients suffer similar marked weight/LBM loss, we must consider that significant calorie/protein delivery will be required to restore this lost LBM and QoL. This is supported by the aforementioned seminal metabolism studies showing that the average TEE in the second week of ICU stay was 47 kcal/kg/day in sepsis and 59 kcal/kg/day in trauma [12] (Table 1). This is well beyond what most units deliver to recovering ICU patients; however, these are actual measured metabolic requirements of patients as they recover, and with new early ICU mobility programs this delivery of increased energy in the recovery phase may be vital.

These data demand that we ask whether it is possible our patients have been unable to recover their QoL post ICU for months to years due to our lack of understanding of their fundamental metabolic needs in different phases of illness? For example, the need for additional protein intake has been well described by Hoffer and Bistrian [32–34] in a number of recent publications questioning whether it is actually “protein-deficit” and not calorie deficit that is important to improving outcome in critical illness.

### Personalizing nutrition following discharge to optimize recovery

Finally, we must ask ourselves whether patients leaving our ICUs will be able to consume adequate calories and protein to optimally recover? I think experience has taught us in most cases that the answer is certainly not! Recovering patients, especially elderly individuals, are challenged by decreased appetites, persistent nausea, and constipation from opiates, and lack of education about how to optimize their diet [18]. In ICU patients in the week following extubation, an observational study demonstrated an average spontaneous calorie intake of 700 kcal/day and the entire population studied consumed < 50% of calorie/protein needs for 7 days [35]. It also emphasizes the importance of closely observing food intake in postoperative patients. To address this, a large body of data demonstrates that oral nutrition supplement (ONS) must become fundamental in our post-ICU and hospital discharge care plan. Meta-analysis in a range of hospitalized patients demonstrates that ONS reduces mortality, reduces hospital complications, reduces hospital readmissions, shortens the length of stay, and reduces hospital costs [36–39]. A large hospital database analysis of ONS use in 724,000 patients matched with controls not receiving ONS showed a 21% reduction in hospital LOS and that for every $1 (US) spent on ONS, $52.63 was saved in hospital costs [40]. Finally, a very recent large randomized trial of 652 patients and 78 centers studied the effect of high-protein ONS with β-hydroxy β-methylbutyrate (HP-HMB) versus placebo ONS in older (≥ 65 years), malnourished (Subjective Global Assessment (SGA) class B or C) adults hospitalized for congestive heart failure, acute myocardial infarction, pneumonia, or chronic obstructive pulmonary disease over 90 days in the hospital and post-hospital period [41]. The data demonstrated that high-protein HP-HMB reduced 90-day mortality by ~ 50% relative to placebo (4.8% vs 9.7%; relative risk 0.49, 95% confidence interval (CI) 0.27 to 0.90; *p* = 0.018). The number needed to treat to prevent one death was 20.3 (95% CI 10.9 to 121.4) [41]. This trial was key as it was the first large multicenter randomized controlled trial to confirm the extensive data from smaller trials demonstrating a similar beneficial effect.

### Role of specific anabolic/anti-catabolic agents, vitamin D, and microbiome/probiotics in recovery

**Fig. 5:**
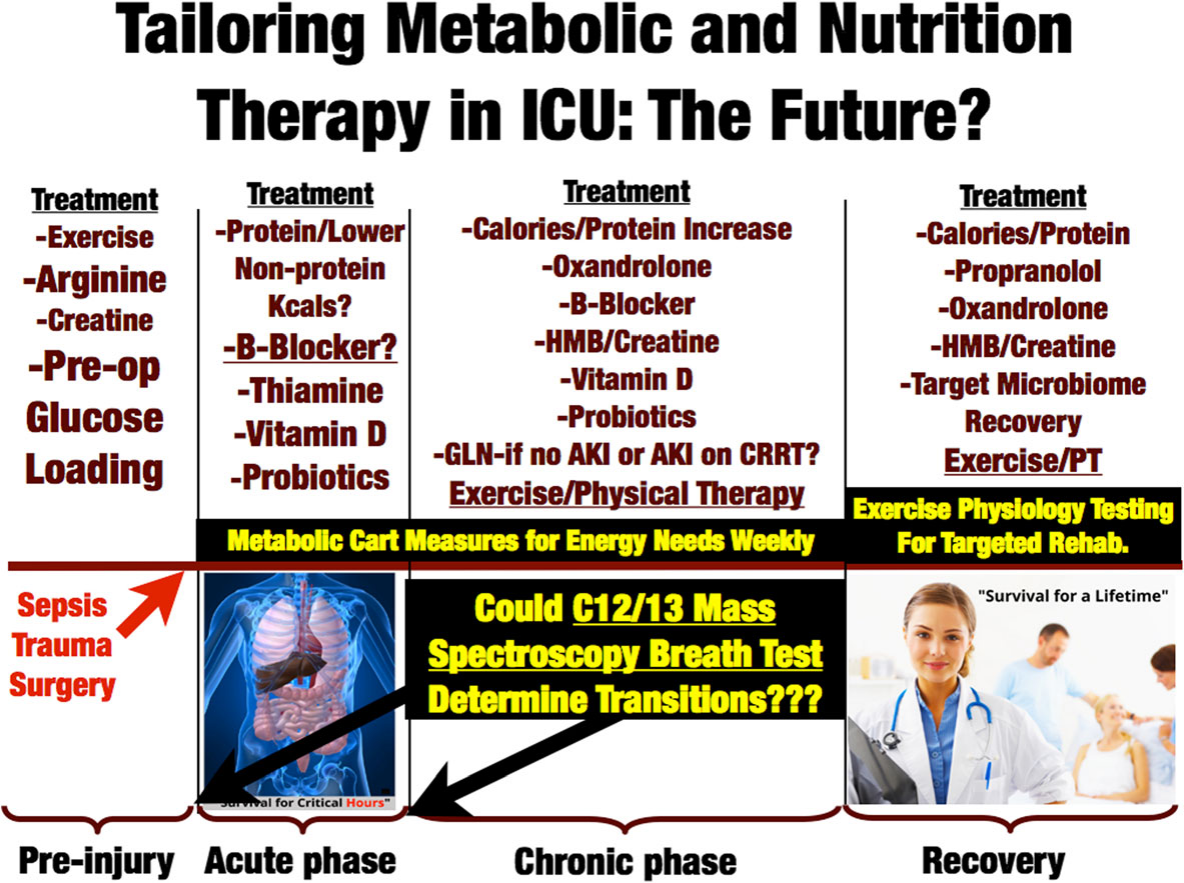
Targeted nutritional and metabolic therapy in critical illness. Adapted from [18]

The data from the large ONS trial using HMB [41] and recent data emphasize that anabolic/anti-catabolic interventions, such as propranolol, oxandrolone, and other agents targeted at restoring lean muscle mass (such as HMB), may be vital in optimal recovery and survival from critical illness [42]. As shown in Fig. 5, targeted nutrition with adequate protein delivery and “muscle-recovery targeted” agents when combined with exercise will likely play a vital role in improving survival and recovery of QoL post ICU [21]. Figure 5 also shows the emerging key role for vitamin D to reduce mortality in vitamin D-deficient ICU patients (as shown in the recent *JAMA* paper by Amrein et al. [43]), as was reviewed in expert detail recently by Christopher [44]. Further, new data indicate that thiamine deficiency occurs in up to 35% of septic shock patients [14]. This recent randomized, double-blind, controlled trial administered 200 mg thiamine to patients with septic shock and elevated lactate. Although administration of thiamine did not improve survival in the overall group of patients with septic shock, in thiamine-deficient patients a statistically significant decrease in mortality over time for those receiving thiamine was observed (*p* = 0.047), as well as reduced lactate at 24 hours [14]. Finally, new data expanding our understanding of the microbiome in the ICU and “dysbiosis” therapies including probiotics and fecal microbiota transplantation (FMT) have recently been reviewed by our group [45]. A summary of these interventions and their proposed timing is described in Fig. 5.

### My personal experience with optimizing nutrition delivery during recovery following acute illness

As described previously [21], I have personally experienced critical illness and major surgical interventions throughout my life as a result of complications of ulcerative colitis and > 20 subsequent surgeries. Thus, recovery from ICU and surgery is a part of my daily life. I faced recovering from ICU and surgery once again in summer 2014, when I was in perhaps the best physical condition of my life, only to acutely suffer a major bowel obstruction leading to massive bowel edema and an operation that led to a brief ICU stay and a prolonged hospital stay postoperatively. During this 23-day postoperative stay I lost 20 kg of body weight (quite similar to the total weight loss of the Minnesota Starvation Study—only over a much shorter time-frame). At discharge, I had lost significant LBM and was not able to walk down the hospital hallway without being short of breath. As I had found following previous major operations and subsequent weight loss episodes, I needed to consume 4000–5000 kcal/day for ~ 18 months, exercise 5 days/week, and take 2.0 g/kg/day of protein to regain the strength, QoL, function, and weight I had enjoyed prior to surgery. In addition, over 30 years of personal experience I have refined a daily regimen of anabolic and anti-catabolic supplements as presented in Table 3. Again, I personally was struck how accurate and vital the data from the Minnesota Starvation Study is today for both our patients and even myself to optimize recovery.

**Table 3:** Post-ICU/postoperative targeted rehabilitation nutrition program (PEW’s daily program)

Exercise	Run and weight train 5 days/week
Nutrition	4000–5000 kcal/day
Calories	2 g/kg/day
Protein (whey, eggs)	(~ 2.0 g/kg body weight)
Supplements	
Branch chain amino acids	10 g/night
HMB	3 g/day
Vitamin D	2000 IU/day
Fish oil	2 g/day
l-Carnitine	Daily
Stress B multivitamin complex	Daily
Alpha lipoic acid	600 mg BID
DHEA	100 mg/BID
β-alanine	4–5 g/day
Creatine	5 g/day first 6–12 months post ICU (or longer for potential benefits on cognition and muscle strength)
Glutamine	10 g BID first 3–6 months post ICU

Note: This is the author’s personal recovery program developed over 30 years of personal experience with illness, surgery, and ICU recovery. It is not suggested that this program is ideal for all recovering individuals. It is only meant as a suggestion to consider in recovery. Readers are encouraged to email the author (Paul.Wischmeyer@Duke.edu) with specific questions and evidence for particular elements of the program

*BID* twice daily, *HMB* β-hydroxy β-methylbutyrate

## Conclusions

We need to consider basic metabolism and our historic understanding of starvation and recovery to employ targeted nutritional care for our critically ill patients. If we are to optimize patient outcomes and start creating “survivors and not victims” we must realize that one-size nutrition and one calorie delivery “does not fit all”. It is clear our patients’ nutritional needs change over the course of illness. Further, the presence of preexisting nutritional risk, such as that defined by the NUTRIC Score or sarcopenia (even low BMI < 25 as described by our recent published TOP-UP trial of supplemental PN [30]) should guide how we feed our patients, with high-risk malnourished patients getting more aggressive early calorie (~ 25 kcal/kg) and protein delivery via early EN and/or PN. Lower risk patients likely need lower early calories ~ 15 kcal/kg/day with adequate protein (~ 1.2 g/kg/day) as supported by the 2016 SCCM/ASPEN Guidelines. Early enteral nutrition during the acute phase should attempt to correct micronutrient/vitamin deficiencies, deliver adequate protein, and moderate nonprotein calories in well-nourished patients, as in the acute phase they are capable of generating significant endogenous energy. Post resuscitation, increasing protein (1.5–2.0 g/kg/day) and calories are needed to attenuate LBM loss and promote recovery. Malnutrition screening is essential and parenteral nutrition can be safely added following resuscitation when enteral nutrition is failing based on pre-illness malnutrition and LBM status. Following the ICU stay, significant protein/calorie delivery for months or years is required to facilitate functional and LBM recovery, with high-protein oral supplements being essential to achieve adequate nutrition. To better understand the nutrition delivery required in the post-ICU period, we must all take a moment to read and revel in the defining achievement that is the Minnesota Starvation Study and learn from its landmark lessons. Most important among these is that even healthy subjects require significant calories (typically > 3000-4000 kcal/day) to recover from massive weight and LBM loss, such as occurs following critical illness (or even major surgery). How will many of our care protocols, or our patients, acknowledge or achieve this well-described goal? Is it possible that this lack of understanding of caloric and protein need in recovery has led to the extremely poor long-term outcomes and QoL that follows ICU care? Only time and further research will tell for sure. But, as always, this increase in calorie delivery should be targeted with objective data when possible via use of improved metabolic cart technology. In the future, great promise seems to exist for bedside ^13^C/^12^C breath carbon ratio mass spectroscopy [46, 47] to assist in direct objective measurement of overfeeding and underfeeding. Finally, we must learn to target and incorporate nutritional therapies such as vitamin D, probiotics, and anabolic/anti-catabolic agents to optimize our patients’ chance to survive and thrive against all evolutionary odds. We have long known Mother Nature does not want our ICU patients to win this war and become “survivors … and not victims”. But to begin winning the war on long-term ICU outcomes and give our patients back the lives they came to us to restore, we must ensure our patients are getting the right nutrition, in the right patient, at the right time!
